# Polyphyllins ameliorate imiquimod-induced psoriatic skin barrier disruption and lesions in mice by inhibiting inflammatory infiltration with accelerated recovery effects

**DOI:** 10.3389/fimmu.2026.1821182

**Published:** 2026-07-27

**Authors:** Siqi Ren, Yang Yang, Huiwen Zhang, Jingmin Tian, Xiao Li, Ying Zhang, Wuzhou Wan, Ping Li, Jiannan Ma, Jianye Wang, Qianghua Quan

**Affiliations:** 1 School of Pharmacy, Inner Mongolia Medical University, Hohhot, China; 2 R&D Department, Yunnan Baiyao Group Co., Ltd., Kunming, Yunnan, China

**Keywords:** inflammation, macrophage infiltration, polyphyllins, psoriasis, skin barrier

## Abstract

**Background:**

Psoriasis is a chronic inflammatory skin disorder that significantly compromises patients’ quality of life. While dexamethasone (DEX) is effective as a first-line treatment, its adverse effects highlight the need for alternatives. Effective therapies for prolonging clinical remission and reducing relapse rates remain limited. Our previous study demonstrated that Polyphyllin H (PPH) and PPH-enriched Rhizoma Paridis extract (RPE) possess great anti-inflammatory efficacy *in vitro*, with polyphyllins also improving skin barrier integrity. However, their therapeutic efficacy and safety in chronic inflammatory conditions remain unexplored.

**Methods:**

In this study, we established an imiquimod (IMQ)-induced psoriasis model in BALB/c mice, in which psoriatic lesions were first induced by IMQ application and subsequently monitored for their regression upon IMQ withdrawal and treated with RPE, Polyphyllin H (PPH), and DEX. Psoriasis Area Severity Index (PASI) scores, pathohistological analysis and inflammatory cytokines expression of psoriasis-like mice were measured in different groups.

**Results:**

Over a 12-day observation period, RPE significantly ameliorated psoriasis-like lesions, facilitating lesion remission. Histopathological analysis revealed that RPE prevented epidermal thickening with reduced Ki67 expression. Furthermore, PPH, the main active component of RPE, significantly improved PASI scores and reduced epidermal thickening in psoriasis-like lesions. Mechanistically, both PPH and RPE suppressed macrophage infiltration and downregulated key inflammatory cytokines associated with psoriasis.

**Conclusion:**

In conclusion, our findings establish polyphyllins as promising anti-inflammatory agents that promote psoriasis lesion resolution while offering a safer alternative to corticosteroids. Our study underscores the therapeutic potential of polyphyllins for psoriasis management and warrants further investigation into their clinical applicability.

## 1 Introduction

Psoriasis exhibits a global prevalent distribution, with a prevalence of 2-3%. (1) Psoriasis is an immune-mediated chronic inflammatory skin disease characterized by erythematous lesions covered with silvery-white scales, often accompanied by systemic inflammation. (2) Its pathogenesis involves a complex interplay between inflammatory cytokines and skin barrier dysfunction. (3–5) Key pathological hallmarks includes abnormal proliferation/differentiation of keratinocytes, hypervascularization, and infiltration of inflammatory cells, such as macrophages, T-cells, dendritic cells, and natural killer cells. (2, 6–8) The interleukin-17 (IL−17)−mediated inflammatory cascade recruits and activates immune cells, which create a pro−inflammatory microenvironment and release cytokines such as interleukin-1β (IL-1β), tumor necrosis factor-α (TNF-α), and interferon−γ (IFN-)γ. These cytokines induce keratinocyte hyperproliferation, leading to epidermal thickening, scaling, and barrier dysfunction. This disrupted barrier further fuels cytokine release, forming a self-perpetuating inflammatory cycle. (9–12).

Psoriasis follows a cyclical course, alternating between acute exacerbations and remission. (1) The acute phase is primarily driven by overactivation of Th17 cells and the IL 23/IL 17 axis, leading to epidermal hyperplasia, neutrophil infiltration, and angiogenesis. During remission phase, although clinical improvement occurs, low grade inflammation persists. (13) Maintenance therapy during remission, such as continuous use of calcipotriol/betamethasone foam, has been shown to reduce relapse frequency and prolong remission. (14) The Guidelines for the Diagnosis and Treatment of Psoriasis emphasize that maintenance therapy aims to consolidate therapeutic responses, prevent relapse, and mitigate disease burden. (15) However, long-term or high-dose use of glucocorticoids like dexamethasone (DEX) impairs skin barrier integrity and often leads to rapid relapse after withdrawal, while conventional moisturizers have limited efficacy. (16–18) This highlights the urgent need for novel therapeutic strategies specifically for remission-phase management.

The exploration of anti-inflammatory natural compounds presents a promising avenue for psoriasis treatment due to their high safety profiles and potential for chronic disease management. (19, 20) Among them, Rhizoma Paridis, a traditional Chinese medicine, has long been used for infections, inflammatory diseases, and cancer, with steroidal polyphyllins as its primary bioactive constituents. (21) Polyphyllins exhibit excellent anti-inflammatory pharmacological activity. Polyphyllin I suppresses NF-κB activation and inhibits the release of interleukin-8 (IL-8), interleukin-6 (IL-6), and TNF-α, which are key inflammation mediators in psoriasis. (22, 23) Polyphyllin VII inhibits inflammatory responses in the lipopolysaccharide (LPS)-induced RAW264.7 cells and multiple animal models by downregulating MAPK and NF-κB pathways. (24) Our previous studies have shown that Polyphyllin H (PPH) and PPH-enriched Rhizoma Paridis extract (RPE) have anti-inflammatory and antioxidant activities in LPS-induced macrophages and keratinocytes, attenuating reactive oxygen species (ROS) production via NRF2/HO-1 pathway activation, inhibiting NF-κB nuclear translocation, and suppressing pro-inflammatory cytokine expression. Furthermore, PPH has been shown to enhance skin barrier integrity, suggesting its potential for managing inflammatory dermatoses. (25) However, despite its promising pharmacological profile, polyphyllins remain underexplored in psoriasis translational research. Moreover, their safety and therapeutic efficacy in preclinical psoriasis models remain largely unverified.

To bridge this gap, we aimed to elucidate the therapeutic potential of PPH in psoriasis by investigating its effects on skin barrier restoration and inflammatory modulation. We established an imiquimod (IMQ)-induced psoriasis mouse model, in which psoriatic lesions were first induced by IMQ application and subsequently monitored for their natural regression upon IMQ withdrawal. This model mimics the residual low-grade inflammation characteristic of the psoriasis remission phase and provides a clinically relevant platform for developing maintenance therapies. Using this model, we assessed whether PPH and RPE could accelerate lesion resolution and enhance skin recovery, thereby providing a scientific basis for the development of novel therapeutic strategies for psoriasis management.

## 2 Materials and methods

### 2.1 Reagents

RPE was sourced from Yunnan Baiyao Group Co., Ltd (Kunming, China). PPH was purchased from Sarbio (SP5270, Beijing, China). Imiquimod cream was obtained from Sichuan Mingxin Pharmaceutical Co., Ltd (Sichuan, China). Dexamethasone was produced by Shanghai yuanye Bio-Technology Co., Ltd (S17003, Shanghai, China). ELISA kits of Mouse TNF-α (BN50578), IL-10 (BN50534), IL-17A (BN50539), IL-1β (BN50543) were purchased from BIORIGIN; mouse IFN-γ (RX203097M) ELISA Kit was purchased from RUIXIN BIOTECH. SweScript All-in-One RT SuperMix was purchased from Servicebio (G3337-50, Wuhan, China). Tissue Protein Extraction Kit was produced by CWBiotech Inc. Abs against F4/80 (ab300421), and Ki67 (ab279653) were produced by Abcam (Waltham, MA, USA). Goat Anti-Rabbit IgG and Goat Anti-Mouse lgG were purchased from Abways (Shanghai, China).

### 2.2 Mouse model of psoriasis induced by imiquimod

BALB/C male mice 7–8 weeks of age, with a weight of 20-25g, obtained from Inner Mongolia Medical University (Inner Mongolia, China) were used in this experiment. Mice were acclimated in a specific pathogen-free condition with constant temperature (22 ◦C) and provided with water and food ad libitum for 7 days before experiments. The experimental operations were conducted in accordance with the regulations of the Ethics Committee for Laboratory Animals of Inner Mongolia Medical University.

Mice were randomly assigned to seven groups (n = 8 per group): Control, Imiquimod (IMQ), Dexamethasone (DEX), high-dose RPE (RPE-H), low-dose RPE (RPE-L), high-dose PPH (PPH-H) and low-dose PPH (PPH-L) groups. An area of 2 cm × 3 cm on the back of the mice was shaved with surgical clippers on the last day of adaptive housing. The control group was treated with saline (125 μL/d), while each mouse in other groups was topically administered with 62.5 mg/d of 5 w/w% imiquimod cream on the shaved back for 5 days to induce the psoriatic models, as described previously. (26) From day 6, the RPE-H group and RPE-L group were received topical RPE treatment at doses of 0.05 w/w% and 0.01 w/w%, respectively, with 125 μL twice daily. The PPH-H group and PPH-L group were treated with PPH at doses of 0.015 w/w% and 0.003 w/w%, respectively, with 125 μL twice daily. These doses were selected based on our previous *in vitro* study (25) and converted to *in vivo* doses using standard preclinical dose translation. The DEX group received topical dexamethasone 0.03 w/w% (dose-equivalent to 1.5 mg/kg/day) as a positive control, a dose within the reported range for IMQ-induced psoriasis models (27). All animals were maintained without additional IMQ intervention to allow spontaneous resolution of inflammation. After 7 consecutive days of treatment, all mice were euthanized, and dorsal skin samples were collected for further analysis.

### 2.3 PASI score

The PASI score system referred to the report, (28) in which erythema, thickness, and scale were measured on a 0–4 scale (none, slight, moderate, severe, extremely severe), and the total PASI score (erythema plus scaling plus thickening) was measured on a 0–12 scale, reflecting the severity of psoriasis lesions and evaluating the recovery effect of RPE and PPH in the IMQ-induced psoriasis mice model as shown in **Supplementary Table S1**. We performed serial assays and recorded them within 12 days of the psoriasis mouse model establishment and administration of treatment.

### 2.4 Histopathology analysis

Animal skin samples were collected, and fixed with 4% para­formaldehyde for 24 h. Briefly, the tissues were embedded in paraffin and serial sectioned into 4 μm-thick skin sections, followed by staining with hematoxylin and eosin (H&E). A microscope (HALO, KFBIO, China) was used to observe the sections. Images were captured at ×100 (10×10 objective) and ×400 (40×10 objective) magnifications. For immunofluorescence, 4 μm-thick skin serial sections were deparaffinized, hydration, treated with sodium citrate buffer at pH 6.0 for antigen retrieval, and incubated with the primary Ab (diluted with 5% normal goat serum) overnight at 4 °C. The primary antibodies used were rabbit anti-Ki-67 (dilution 1:200, abcam, ab279653) and rabbit anti-F4/80 (dilution 1:200, abcam, ab300421). After washing, sections were incubation with the goat anti-rabbit IgG (H&L) secondary Ab (dilution 1:7000, AB0151, Abways, Shanghai, China) at RT for 2 h. Subsequently, the cells mounted with a hard-set mounting medium with 4’, 6-diamidino-2-phenylindole (DAPI) were then imaged. Images were captured at ×300 (30×10 objective) magnifications. All procedures were performed according to the manufacturers’ guidelines. ImageJ software (version 1.53a) was used to quantify fluorescence intensity from random fields.

### 2.5 ELISA analysis

The expression of IL-10, IL-17, TNF-α, IFNγ, and IL-1β in the supernatant of mouse skin homogenate were measured using mouse IL-10, IL-17, TNF-α, IL-1β ELISA kits (Biorigin, Beijing, China). According to the manufacturer, the detection ranges were 3.12–200 pg/mL for IL-10, 4.68–300 pg/mL for IL-17, 15.62–1000 pg/mL for TNF-α, and 3.12–200 pg/mL for IL-1β; the intra−/inter−assay coefficients of variation (CV) were all <10%. Mouse IFN−γ was measured using Mouse IFN−γ ELISA kit (RUIXIN BIOTECH, Quanzhou, China), with a detection range of 7.81–500 pg/mL and intra−/inter−assay CV <10%. All assays were performed according to the manufacturers’ instructions. Briefly, each 10 μL supernatant from the control and experimental groups and 40 μl sample diluents were added to the ELISA plate and incubated for 30 min at 37 ◦C. The plate was washed with washing buffer five times. Then enzyme standard reagent (50 μL) was added and incubated for 30 min followed by washing four times. Finally, chromogenic agent A (50 μL) and chromogenic agent B (50 μL) were added into each well in turns and incubated in the dark for 10 min. Then the stop solution (100 μL) was added to each well. The absorbance at 450 nm was measured within 15 min by using a microplate reader.

### 2.6 Total RNA extraction and qPCR analysis

Total RNA was extracted from mouse dorsal skin tissues using TRIzon Reagent (CW0580S, CWBIO, Jiangsu, China) and RNA concentration was measured with a Multifunctional Enzyme Labeler (SpectraMax i3, Molecular Devices, SV, USA). PrimeScriptTM reagent Kit with gDNA Eraser (Takara, Kusatsu, Japan) was used to synthesize cDNA. qPCR was performed using SweScript All-in-One RT SuperMix (G3337-50, Servicebio, Wuhan, China) on a Applied Biosystems™ 7500 Real-Time PCR machine (Prsm7500, Applied Biosystems, USA). The primers used were synthesized by Sangon Biotech (Shanghai, China) (**Supplementary Table 3**). GAPDH housekeeping gene was used as an internal control. Transcript levels of target genes were calculated using the 2−ΔΔCt method.

### 2.7 Randomization, blinding and inclusion criteria

Inclusion criteria for animals were male BALB/C mice, 7–8 weeks of age, body weight between 20–25 g, with no visible skin lesions or systemic illness before the experiment. Exclusion criteria were death during the experiment, accidental injury, or any sign of infection. Animals meeting any exclusion criterion were removed from the analysis.

The experiment was blinded: the investigator responsible for drug administration was different from the one performing outcome assessments, and the assessor was kept unaware of group allocation. Mice were randomly assigned to groups. From each group three animals were further randomly selected for histological and molecular assays. This sample size complies with the 3R principle (Reduction) by maximizing data output per animal. A portion of tissue samples were used for H&E and IHC, and the remainder were used for qPCR and ELISA. As an exploratory study, a sample size of three is acceptable for generating descriptive evidence and identifying preliminary trends. (29) Each experiment was repeated at least three times, and reproducible results were obtained. Representative data are shown in the figures.

### 2.8 Statistical analysis

GraphPad PRISM 10 software (GraphPad Software, Boston, USA) was used for all statistical analyses performed in this study. Group differences were analyzed by one-way ANOVA followed by Tukey’s Multiple Comparisons.

## 3 Results

### 3.1 RPE ameliorates IMQ-induced psoriatic skin lesions in mice with accelerated recovery effect

**Figure 1:**
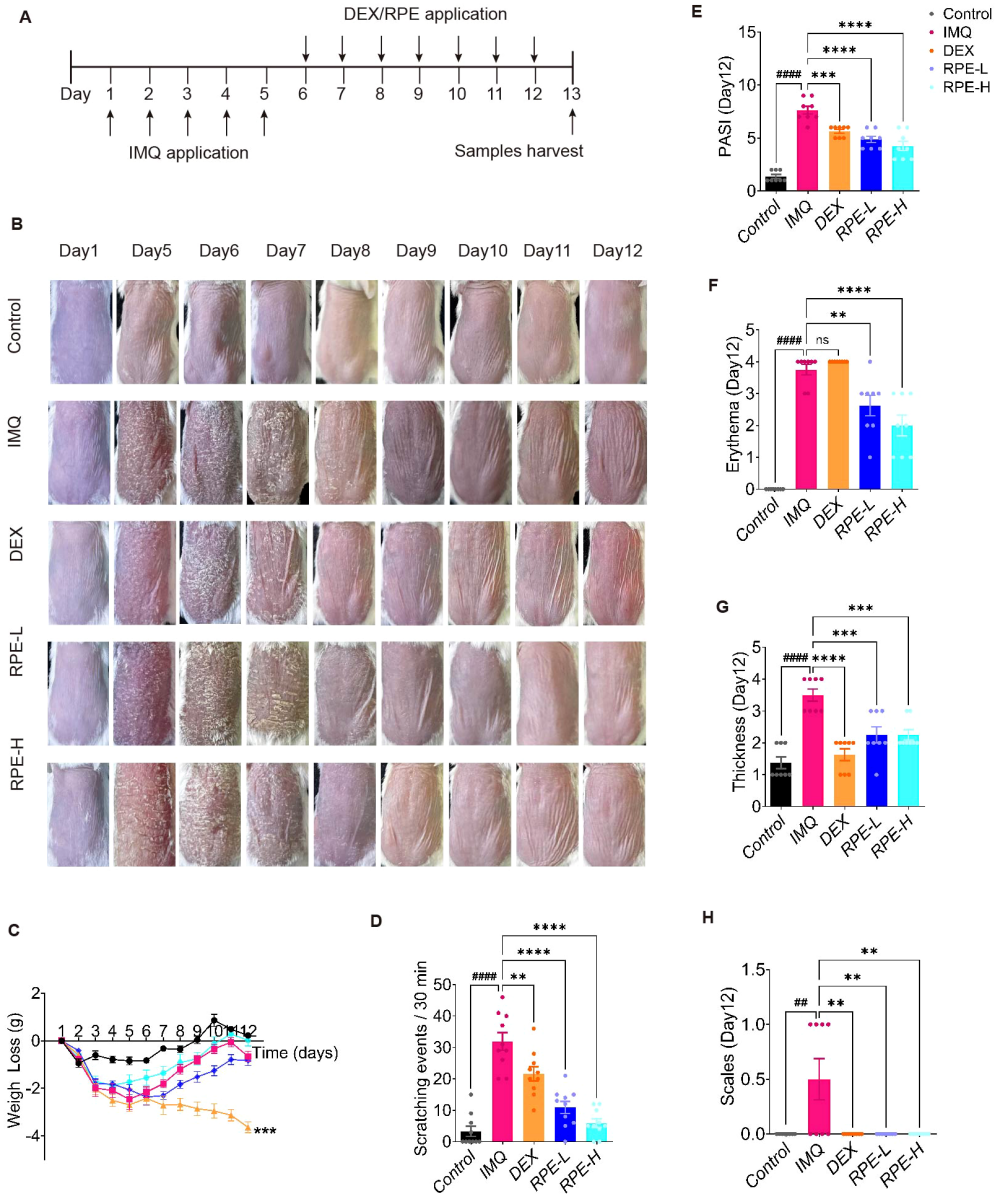
Assessment of skin appearance and psoriasis area and severity index (PASI) in a mouse model. **(A)** Schematic representation of the experimental design, illustrating the timeline of psoriasis-like skin induction with imiquimod (IMQ) and the drug treatment in BALB/c mice. **(B)** Representative images showing skin appearance on day 1, 5, and 6-12. **(C)** Body weight change during the experiment. **(D)** Quantification of scratching events on day 12. PASI scores on day12, including scoring of total PASI **(E)**, skin erythema **(F)**, thickness **(G)**, and scales **(H)**. For each day, statistical significance was analyzed by one-way ANOVA followed by Tukey’s Multiple Comparisons. Data plotted as mean ± standard error (n = 8 animals/group). Comparisons with the control group, ^###^p < 0.001, ^####^p < 0.0001; Comparisons with the IMQ group, **p < 0.01, ***p < 0.001, ****p < 0.0001. ns, not significant.

To assess the effects of RPE on psoriasis remission, we established an IMQ-induced psoriasis mouse model, in which psoriatic lesions were first induced by IMQ application and subsequently monitored following IMQ withdrawal. The timeline of IMQ, DEX, and RPE application on BALB/c mice was shown as **Figure 1A**. As shown in **Figure 1B**, for five consecutive days of modeling, no abnormalities were observed in the control group, while the skin of mice in all the groups except the control group showed obvious skin damage symptoms of erythema, scaling, and skin thickening. Following IMQ withdrawal, all groups of modeling mice showed different degrees of reduction of skin lesions, indicating successful modeling and simulating the spontaneous remission of psoriasis.

To quantify disease severity and treatment efficacy, we monitored dorsal skin appearance, body weight changes, and scratching behavior daily, alongside scoring psoriatic severity using a modified PASI scale. (28) RPE-treated mice exhibited progressive PASI score improvement over seven consecutive days, with a significant reduction observed on day 8, indicating a potentially earlier onset of therapeutic efficacy compared to the DEX group (**Supplementary Table 3**). Notably, the total PASI scores of RPE groups were highly significantly decreased on day 12 compared to the IMQ group (p<0.0001), and the DEX group was significantly decreased (p<0.001, **Figure 1E**; **Supplementary Table 3**). Specifically, in terms of erythema, the RPE-H group exhibited significant improvement, with scores decreasing to 2 (slight) by day 12, whereas the DEX group failed to produce a comparable reduction (**Figure 1F**; **Supplementary Table 4**). For skin thickness, RPE-L group decreased significantly starting on day 11, while in the DEX group it decreased on day 12 and was lower (**Figure 1G**; **Supplementary Table 5**). Scaling scores fell to 0 (none) on day 12 in all treatment groups, but the RPE-H group achieved it by day 11 (p < 0.05, **Figure 1H**; **Supplementary Table 6**).

In addition to lesion resolution, pruritus reduction serves as a key indicator of psoriasis recovery during the remission phase. (30) Our results showed that compared to the IMQ group, scratching events were significantly reduced in the RPE-treated groups (p < 0.0001) and DEX group (p < 0.01) (**Figure 1D**), suggesting a potential advantage for itch relief with RPE treatment. Body weight trends serve as an indirect indicator of systemic treatment safety. While mice in the DEX group exhibited significant weight loss compared to the IMQ group (p < 0.001), RPE-treated mice maintained stable body weight, suggesting a more favorable safety profile for RPE under the present experimental conditions (**Figure 1C**).

In conclusion, both RPE and DEX treatments could alleviate IMQ-induced skin lesions. Notably, RPE showed a trend toward accelerated lesion resolution, improving skin barrier recovery, and shortened overall recovery time in this experimental setting. Additionally, RPE demonstrated pruritus relief and a favorable safety profile, suggesting that RPE may be a potential candidate for psoriasis remission-phase management.

### 3.2 RPE inhibits epidermal hyperplasia in the IMQ-induced psoriasis mice model with reduced Ki67 expression

**Figure 2:**
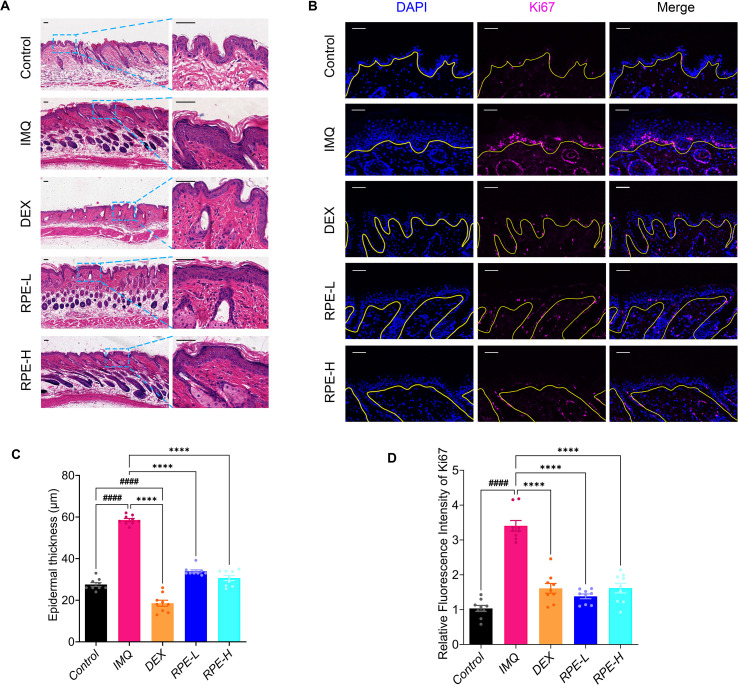
Pathohistological analysis of psoriasis-like skin lesion across different treatment groups. **(A)** Representative H&E staining of dorsal skin sections from mice. **(B)** Immunofluorescence results of Ki67. The nuclear regions were stained with DAPI. The yellow line indicates the boundary between epidermis and dermis. **(C)** Analysis of epidermal thickness. **(D)** Relative fluorescence intensity analysis of Ki67. Statistical significance was analyzed by one-way ANOVA followed by Tukey’s Multiple Comparisons. Data plotted as mean ± standard error (n = 3 animals/group). Epidermal thickness and fluorescence intensity were measured in three randomly selected fields of view per mouse. Scale bar: 50 µm.

To evaluate the histopathological changes in psoriatic lesions following treatment, H&E staining was performed on dorsal skin samples from each group (**Figure 2A**). The control group exhibited normal skin architecture without pathological abnormalities, whereas the IMQ group showed marked epidermal thickening, characterized by a hyperkeratotic stratum corneum and an expanded spinous layer. RPE and DEX treatment alleviated histopathological alterations. Quantitative analysis revealed a significant increase in epidermal thickness in the IMQ group compared to the control group (p < 0.0001) (**Figure 2C**). Treatment with RPE, as well as DEX, significantly reduced epidermal thickness relative to the IMQ group (p < 0.0001). To further assess epidermal proliferation, Ki67 expression, a marker of keratinocyte hyperproliferation, was analyzed (**Figures 2B, D**). Ki67 was minimally expressed in the control group but significantly upregulated in the IMQ group (p < 0.0001). Both RPE and DEX treatments significantly suppressed Ki67 expression in the basal layer of epidermis (p < 0.0001), indicating a reduction in aberrant keratinocyte proliferation. Notably, mice in the DEX group exhibited excessive epidermal thinning compared to the control group (p < 0.0001), which was not seen in the RPE-L and RPE-H groups. These findings suggest that RPE mitigates IMQ-induced histopathological damage and inhibits epidermal hyperplasia with a favorable safety profile.

### 3.3 PPH enhances skin barrier repair and accelerates psoriasis lesion remission

**Figure 3:**
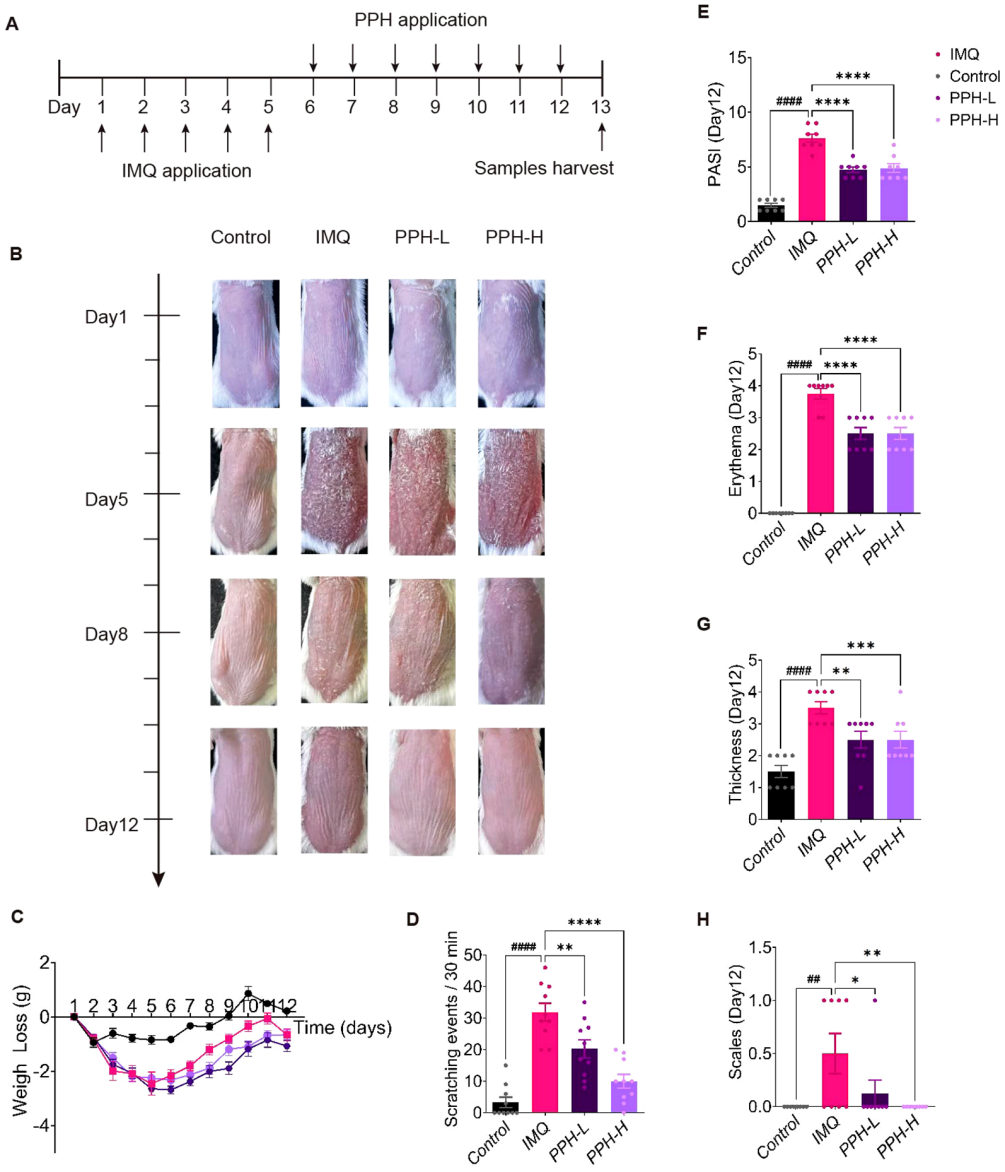
Evaluation of skin appearance and PASI scores in the PPH treatment groups. **(A)** Schematic representation of the experimental timeline. **(B)** Representative images of dorsal skin appearance on day 1, 5, 8, and 12. **(C)** Body weight change during the experiment. **(D)** Quantification of scratching events on day 12. PASI scores on day 12, including scoring of total PASI **(E)**, skin erythema **(F)**, thickness **(G)**, and scales **(H)**. For each day, statistical significance was analyzed by one-way ANOVA followed by Tukey’s Multiple Comparisons. Data plotted as mean ± standard error (n = 8 animals/group). Comparison with the control group, ^##^p < 0.01, ^####^p < 0.0001; comparison with the IMQ group, *p < 0.05, **p < 0.01, ***p < 0.001, ****p < 0.0001.

Given that polyphyllins are the principal bioactive constituents of Rhizoma Paridis, we sought to determine whether PPH plays a pivotal role in accelerating psoriasis lesion recovery. As shown in **Figure 3B**, PPH significantly ameliorated IMQ-induced psoriasis skin lesions with a 7-day treatment. The total PASI scores showed that PPH-intervention mice gradually improved IMQ-induced psoriasis skin lesion for consecutive 7 days compared to the IMQ group (p < 0.0001), and had an onset of effect on day 8 (p < 0.05), along with a highly significant improvement on day 12 (p < 0.0001) (**Figure 3E**; **Supplementary Table 3**). Significant improvements in erythema (**Figure 3F**; **Supplementary Table 4**), thickness (**Figure 3G**; **Supplementary Table 5**), and scales (**Figure 3H**; **Supplementary Table 6**) scores were observed in the PPH groups compared to the IMQ group, with the most pronounced effects in the PPH-H group. The temporal pattern and overall efficacy of PPH were comparable to RPE, suggesting that PPH is a key bioactive component of RPE responsible for psoriasis lesion recovery. Moreover, PPH treatment effectively reduced scratching behavior (**Figure 3D**). Furthermore, body weight remained stable in the PPH-treated groups (**Figure 3C**).

**Figure 4:**
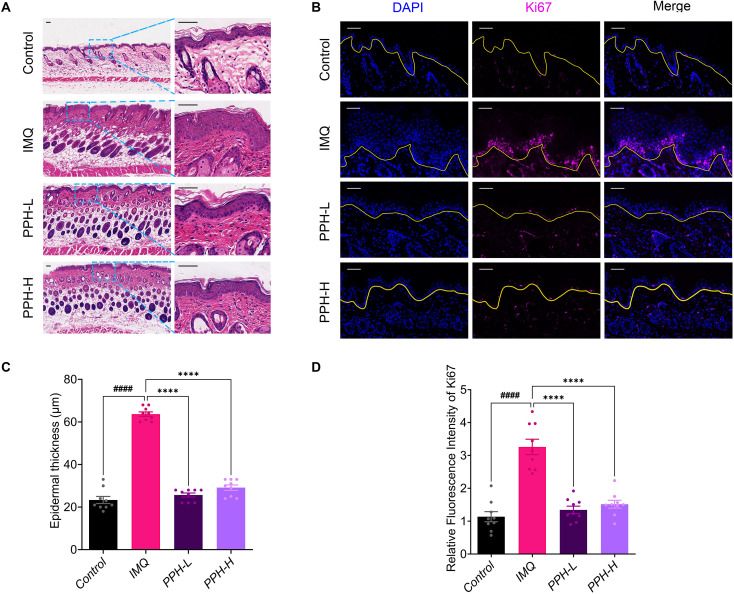
Histopathological analysis of psoriasis-like skin lesion in the PPH treatment groups. **(A)** Representative H&E staining of dorsal skin sections. **(B)** Immunofluorescence results of Ki67. The nuclear regions were stained with DAPI. The yellow line indicates the boundary between epidermis and dermis. **(C)** Quantification of epidermal thickness in different treatment groups. **(D)** Relative fluorescence intensity analysis of Ki67 expression. Statistical significance was analyzed by one-way ANOVA followed by Tukey’s Multiple Comparisons. Data plotted as mean ± standard error (n = 3 animals/group). Epidermal thickness and fluorescence intensity were measured in three randomly selected fields of view per mouse. Comparison with the control group, ^####^p < 0.0001; comparison with the IMQ group, ****p < 0.0001. Scale bar: 50 µm.

Histopathological analysis confirmed that PPH significantly improved IMQ-induced epidermal hyperplasia and skin barrier disruption (**Figure 4**). PPH treatment significantly reduced epidermal thickness compared to the IMQ group (p < 0.0001, **Figures 4A, C**) and suppressed Ki67 expression (**Figures 4B, D**), indicating the inhibition of keratinocyte hyperproliferation. Collectively, these findings validate PPH as the key bioactive component of RPE, effectively mitigating psoriasis-like skin lesions and restoring skin barrier integrity in the IMQ-induced mouse model.

### 3.4 RPE and PPH ameliorate the psoriatic skin barrier disruption and lesions in mice by inhibiting inflammatory infiltration

**Figure 5:**
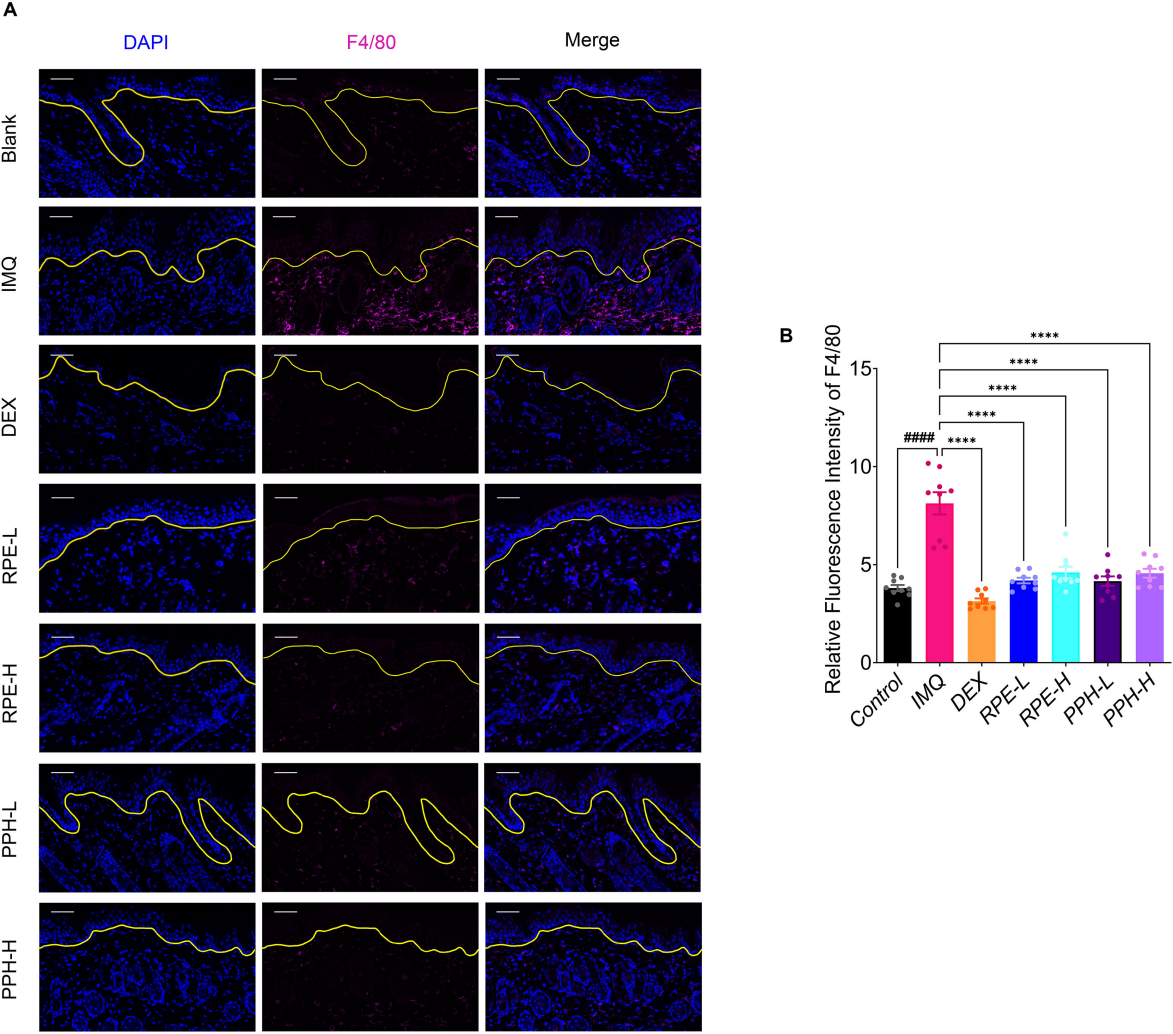
Immunofluorescent staining of F4/80 in psoriasis−like skin lesions of mice in different groups. **(A)** Representative images of F4/80, a macrophage marker. The nuclear regions were stained with DAPI. The yellow line indicates the boundary between epidermis and dermis. **(B)** Quantification of relative fluorescence intensity of F4/80. Statistical significance was analyzed by one-way ANOVA followed by Tukey’s Multiple Comparisons. Data plotted as mean ± standard error (n = 3 animals/group). Fluorescence intensity were measured in three randomly selected fields of view per mouse. Comparison with the control group, ^####^p < 0.0001; comparison with the IMQ group, ****p < 0.0001.

**Figure 6:**
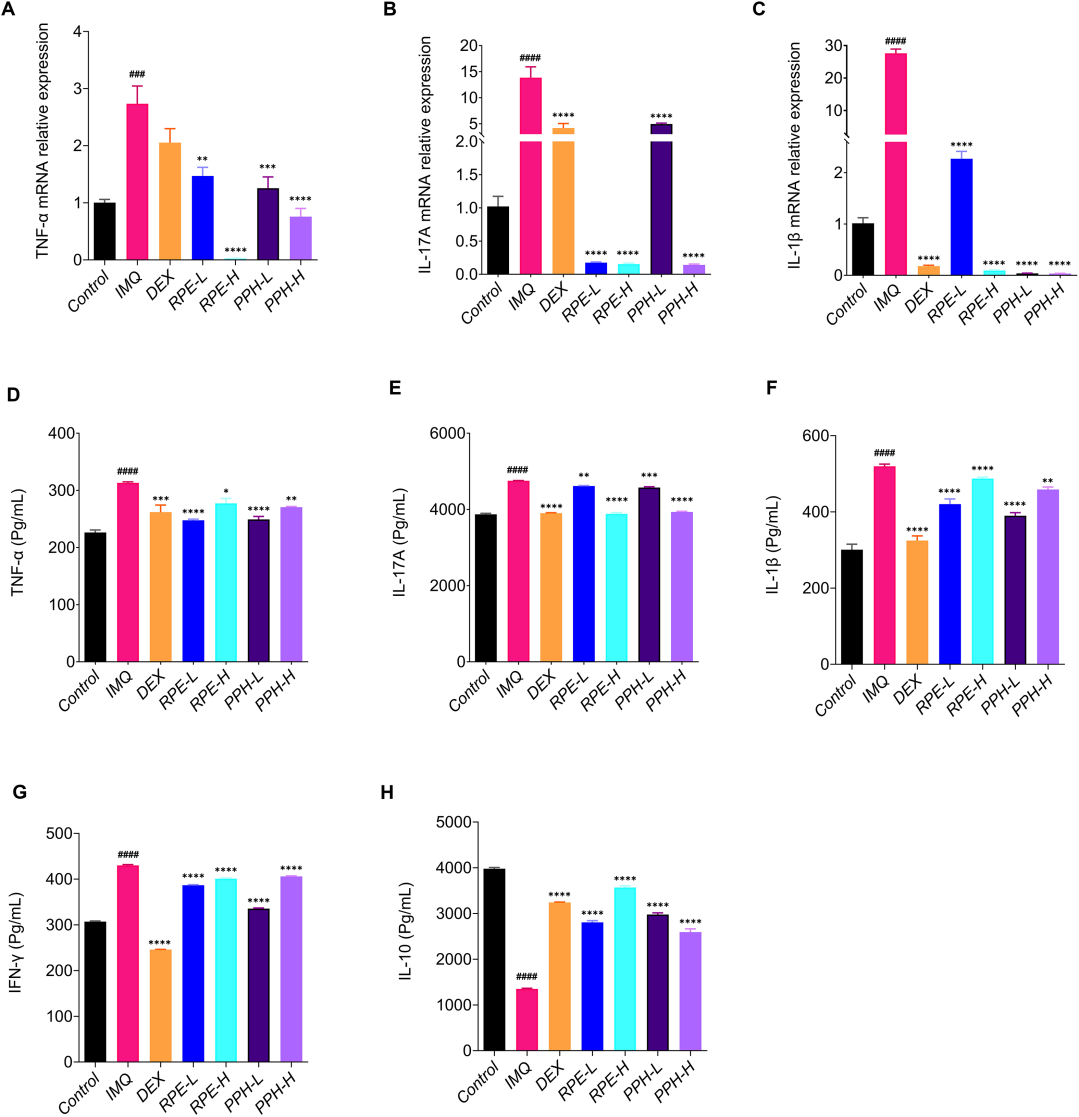
Expression and production of inflammatory mediators in psoriasis-like skin lesion of mice in different groups. **(A–C)**The relative mRNA expression levels of TNF-α **(A)**, IL-17A **(B)** and IL-1β **(C)**. **(B–F)** ELISA analysis of inflammatory cytokine expression: TNF-α **(D)**, IL-17A **(E)**, IL-1β **(F)**, IFN-γ **(G)**, and IL-10 **(H)**. Statistical significance was analyzed by one-way ANOVA followed by Tukey’s Multiple Comparisons. Data plotted as mean ± standard error (n = 3 animals/group). Comparison with the control group, ^####^p < 0.0001; comparison with the IMQ group, *p < 0.05, **p < 0.01, ***p < 0.001, ****p < 0.0001.

Psoriasis is characterized by excessive immune cell infiltration, with macrophages playing a key role in disease progression. (31) F4/80, a well recognized macrophage marker, was used to evaluate macrophage infiltration in psoriatic lesions. In the IMQ-induced model, dermal F4/80 intensity was significantly elevated in psoriatic lesions compared to the control group (p < 0.0001) (**Figure 5**). Treatment with RPE, PPH, and DEX markedly reduced F4/80 levels (p < 0.0001), indicating suppression of macrophage infiltration. Additionally, psoriatic lesions exhibited reduced IL-10 expression and elevated pro-inflammatory cytokines (IL-1β, TNF-α, IFN-γ, and IL-17A). RPE and PPH restored IL-10 levels while significantly downregulating the protein expression of IL-1β, TNF-α, IFN-γ, and IL-17A (**Figures 6D–H**) and the mRNA levels of IL-1β, TNF-α, IL-17A, and IL-6 (**Figures 6A–C**; **Supplementary Figure 1**). Notably, the anti-inflammatory effects in the PPH-L group were comparable to those in the DEX group. Together, these findings demonstrate that PPH and RPE effectively suppress inflammatory infiltration and cytokine expression, mitigating IMQ-induced psoriatic lesions.

**Figure 7:**
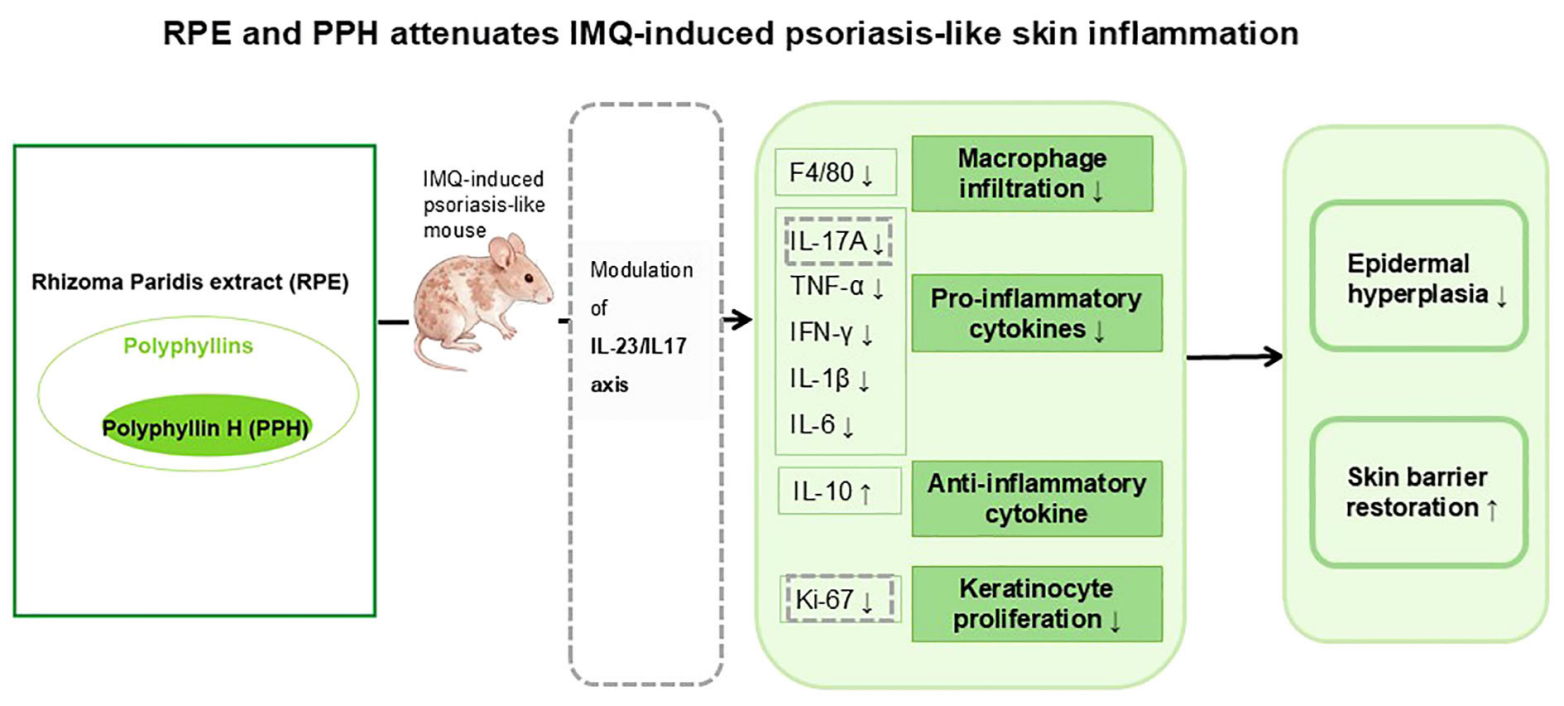
Schematic summary of the observed effects and proposed mechanism of PPH in ameliorating IMQ-induced psoriasis-like skin inflammation. PPH treatment was associated with reduced macrophage infiltration (F4/80), decreased expression of pro-inflammatory cytokines (IL-17A, TNF-α, IFN-γ, IL-1β, IL-6), increased IL-10 expression, and reduced keratinocyte proliferation (Ki-67), correlating with alleviation of epidermal hyperplasia and restoration of skin barrier integrity. Dashed arrows indicate proposed relationships based on correlative evidence; solid arrows indicate experimentally observed effects.

Based on these observations, we summarized the proposed anti−psoriatic mechanism of compound A in a schematic diagram (**Figure 7**), which integrates the observed effects on inflammatory cell infiltration, cytokine expression, keratinocyte proliferation, and epidermal pathology.

## 4 Discussion

Despite advancements in psoriasis treatment, the adverse effects of first-line therapeutics, such as dexamethasone (DEX), pose challenges to long-term disease control. Recent studies have increasingly focused on natural compounds as alternative therapies for psoriasis, given their favorable safety profiles and potential to modulate inflammation and skin barrier function. (20, 32) In this study, we validated PPH as the key active component of RPE, and evaluated the therapeutic efficacy in an IMQ−induced psoriasis remission model, as well as demonstrated its anti-inflammatory and skin barrier-restoring properties. We propose for the first time the potential of polyphyllins for psoriasis treatment, positioning PPH as a promising candidate for psoriasis maintenance therapy.

Psoriasis is a chronic relapsing disease that cycles through acute flares, partial subsiding, and remission, with incomplete immune resolution and residual skin abnormalities persisting during the remission phase. (1, 13) Clinical studies indicate that proper management during this phase can reduce recurrence rates and prolong clinical remission. (33) The IMQ-induced psoriasis mouse model, widely used for evaluating local therapies, mimics the histopathological and immunological features of psoriasis and has been adapted to study disease recrudescence. (26, 34, 35) However, its applicability to the remission phase remains underexplored. Importantly, the IMQ-induced skin inflammation can resolve spontaneously upon withdrawal, and this reversibility provides a direct experimental basis for constructing a remission phase model with an induction, withdrawal, and observation design. Previous studies have reported that in Balb/c mice, after 7 consecutive days of topical application of 5% IMQ followed by withdrawal, the erythema, scaling, and thickening gradually subsided, and by day 15, the mean PASI score had essentially returned to zero. (36) Moreover, the IMQ-induced biphasic mouse model of Terhorst et al. (37) further indicated that day 5 represents the early acute phase with predominant infiltration of neutrophils, monocytes, and monocyte derived dendritic cells, and day 11 represents the late stable phase with increased Langerhans cells and macrophages and a plateauing of inflammation. Based on the above findings, this study adjusted the topical application of 5% IMQ to 5 days, covering the end of the early acute phase, followed by 7 days of withdrawal for continuous observation, aiming to capture the dynamic regression of inflammation after its acute peak. This design maintains initial inflammatory stimulation, avoids skin tolerance and nonspecific injury from prolonged medication, and facilitates observation of residual inflammation in early remission. Consistent with previous findings, IMQ-treated mice exhibited erythema, scaling, epidermal thickening, and inflammatory infiltration, confirming successful disease induction. (26, 38) After IMQ withdrawal, lesions gradually improved, but persistent inflammation remained detectable at both histological and molecular levels, as evidenced by sustained epidermal hyperplasia, immune cell infiltration and elevated pro-inflammatory cytokines (IL-17A, TNF-α), consistent with the sustained inflammatory infiltrates reported by Terhorst et al. (37) Notably, remission in this model represents a down-regulation of inflammation intensity rather than complete resolution, with residual IL-17A, IFN-γ and TNF-α levels remaining above normal, mirroring the clinical observation of residual Th17-related cytokines in human psoriatic remission. (13, 39) These findings validate the model for reflecting persistent low-grade immune activation in the remission stage. However, its acute nature and species differences limit direct translation to human chronic psoriasis (40), and future experiments should incorporate human studys validation or chronic/relapse models to address the limitations. In summary, unlike previous studies that focused primarily on the acute inflammatory phase, the IMQ−induced remission model reasonably recapitulates the resolution of acute inflammation and residual inflammatory features, making it a useful tool for exploring early intervention strategies and screening candidate drugs that may promote resolution and prevent relapse.

Despite its widespread clinical use, DEX therapy is associated with significant limitations, particularly in psoriasis maintenance treatment. Topical corticosteroids often exhibit short-lived efficacy and induce adverse skin reactions, such as irritation, atrophy, and rebound flare-ups upon discontinuation. (41–44) Clinical studies report that prolonged or high-dose glucocorticoid use can lead to irreversible epidermal thinning, highlighting the need for safer alternatives. (45, 46) Consistent with clinical observations, DEX treated mice in our study exhibited significant epidermal thinning and body weight loss, and failed to effectively reduce erythema, reflecting both local and systemic safety concerns. (47, 48) In contrast, PPH and RPE showed favorable efficacy, effectively reduced erythema, alleviated epidermal thickening, without causing noticeable epidermal thinning or weight loss. Furthermore, PPH and RPE significantly reduced pruritus-associated scratching behavior, a common symptom in psoriasis patients that is often inadequately addressed by existing therapies. (49, 50) Under these fixed conditions, RPE and PPH showed comparable efficacy to dexamethasone with a trend toward faster lesion resolution, and all comparative claims are limited to the present experimental setup. These findings suggest that PPH and RPE offer a well-tolerated and effective therapeutic strategy for psoriasis maintenance therapy. Given the short-term nature of this study (12 days), our observations primarily reflect the acute and early remission phase. Accordingly, long-term efficacy and relapse prevention remain open questions that will require extended observation windows. Similarly, safety data in the present work are derived from weight monitoring and histological evaluation of epidermal thickness, which are informative but not exhaustive; a more comprehensive toxicological assessment would be a valuable extension of the current findings in future studies.

Polyphyllins are the main active ingredient group of RPE and have been studied and applied in the field of anti-inflammation. (21–24) Our previous research identified PPH as the major steroidal saponin in RPE, exhibiting potent anti-inflammatory effects via NF-κB pathway inhibition, suppression of pro-inflammatory cytokine expression, and enhancement of antioxidant defenses. (25) These properties contribute to the restoration of skin barrier function and reduction of inflammation-driven keratinocyte damage. In the present study, we confirmed that PPH plays a central role in RPE’s therapeutic effects, as PPH treatment significantly ameliorated psoriasis-like lesions, restored epidermal integrity, and modulated inflammatory responses in IMQ-induced mice. Notably, PPH-enriched RPE is derived from the dried rhizome extract of RP, a plant of the genus Paris within the Liliaceae family, which shares the same family and genus with *Paris polyphylla* Smith var. *yunnanensis* (PPY). However, it has been reported that RP and PPY exhibit compositional differences, with RP containing substantially higher PPH content than PPY, suggesting potential variations in their efficacy. (51) From a clinical perspective, our novel finding suggests the potential for novel drug discovery from plants of the Paris genus.

A complex network of immune cells and molecules plays a key role in the pathogenesis of psoriasis. Studies have confirmed that the pathogenesis of psoriasis can be mediated by pathologic crosstalk between epidermal keratinocytes and immune cells. (52) At the onset of psoriasis, high levels of pro-inflammatory cytokines lead to hyperproliferation of keratinocytes, (53, 54) which results in scaling and keratinization of the skin and damage to the skin barrier. (9–11) Therefore, relieving skin inflammation is a strategic approach to treating psoriasis. Psoriasis is an immune imbalance-mediated autoimmune disease, and that macrophage infiltration and polarization, (11, 31) as well as the inflammatory mediators they secrete, are involved in the onset and development of psoriasis-induced cutaneous inflammation. (26, 55) In our study, RPE and PPH significantly suppressed F4/80 expression, indicating reduced macrophage infiltration, with low-dose PPH exhibiting effects comparable to DEX. Meanwhile, our findings reveal that RPE significantly inhibited the expression of IL-17A, TNF-α, IFNγ, IL-1β, and IL-6, while significantly promoting the expression of the anti-inflammatory factor IL-10, which reflected favorable anti-inflammatory efficacy, and PPH reflected the same results. These observations trend consistently and suggest that RPE and PPH may be associated with attenuation of inflammatory responses and modulation of the immune microenvironment. These data provide valuable clues for understanding the potential action of RPE and PPH The observed reduction in F4/80 expression and the altered cytokine profile could reflect direct modulation of immune cells by RPE and PPH, or alternatively, could be secondary to global improvement of the inflammatory milieu.

Based on these findings regarding inflammatory regulation, we further explored the effect of RPE and PPH on keratinocyte proliferation. Th17 cells and their effector cytokines IL-17A, IL-22, and IL-23 are key drivers of psoriasis. IL-23 promotes Th17 differentiation, while IL-17A and IL-22 synergistically induce keratinocyte hyperproliferation, epidermal thickening, and disordered keratinization. IL-22 mediates acanthosis via signal transducer and activator of transcription 3 (STAT3) signaling, whereas IL-17A upregulates antimicrobial peptides and chemokines and delays terminal differentiation of keratinocytes. Furthermore, keratinocytes also initiate and amplify immune responses by releasing molecules, thereby establishing a self−sustaining inflammatory circuit. (54, 56) As a mature and widely accepted marker of cell proliferation, the expression level of Ki67 is strongly associated with aberrant epidermal growth in psoriasis lesions. (57) In this study, PPH and RPE significantly reduced Ki67 expression in psoriatic skin, indicating their ability to inhibit keratinocyte hyperproliferation. Meanwhile, RPE and PPH significantly downregulated the levels of IL-17A and ameliorated histopathological epidermal thickening and dyskeratosis. Collectively, these results suggest that RPE and PPH may ameliorate epidermal pathology at least partly through suppression of the IL−23/IL−17 axis and reduction of Ki−67−mediated keratinocyte hyperproliferation. Although these mechanistic insights are based on indirect evidence from cytokine and proliferation marker measurements, a limitation attributable to the sample size and experimental scope of this exploratory study, the findings provide a rationale for future investigations to directly assess the effects of RPE and PPH on IL-23/IL-17 signaling. Notably, we acknowledge that the sample size is limited, the observed trends were consistent across three independent animals per group, supporting the reliability of the histological and molecular assays. Given the exploratory nature of this study, future investigations with larger cohorts and dose-equivalence comparisons will help to further validate the efficacy of RPE and PPH, and provide more direct evidence for its mechanism. A schematic summary of the proposed mechanism is presented in **Figure 7**.

In summary, this study demonstrates that PPH effectively ameliorates psoriasis-like lesions by modulating inflammatory cytokine expression, suppressing macrophage infiltration, and restoring epidermal integrity. PPH and RPE exhibit anti-psoriatic efficacy, and facilitated lesion resolution with a favorable safety profile, making them promising candidates for psoriasis maintenance therapy. By monitoring PASI scores over 12 consecutive days, conducting histopathological assessments, and evaluating inflammatory markers, we provide robust evidence supporting PPH as a potential alternative therapeutic agent for psoriasis. These findings pave the way for future research on polyphyllins in clinical applications and highlight their potential in novel psoriasis treatment strategies.

## 5 Conclusion

In this study, we established an imiquimod (IMQ)-induced psoriasis mouse model simulating the spontaneous remission of psoriasis to evaluate the therapeutic potential of PPH-enriched Rhizoma Paridis extract (RPE). Our findings demonstrate that RPE and its key active constituent, Polyphyllin H (PPH), significantly accelerate lesion resolution, restore epidermal barrier integrity, and suppress epidermal hyperproliferation. Mechanistically, PPH and RPE effectively modulate inflammatory responses by suppressing pro-inflammatory cytokines (IL-17A, TNF-α, IFN-γ, IL-1β, and IL-6), enhancing IL-10 expression, and reducing macrophage infiltration. Collectively, these results support polyphyllins as safe, effective, and promising alternatives to corticosteroids for psoriasis management during the remission phase, providing a strong rationale for their future clinical development.

## Data Availability

The original contributions presented in the study are included in the article/**Supplementary Material**. Further inquiries can be directed to the corresponding authors.
