# Supplementary material for: Polyphyllins ameliorate imiquimod-induced psoriatic skin barrier disruption and lesions in mice by inhibiting inflammatory infiltration with accelerated recovery effects
Source: Front Immunol. 2026 Jul 27;17:1821182. doi: 10.3389/fimmu.2026.1821182 (PMC13454872; doi:10.3389/fimmu.2026.1821182)
Supplement: Supplementary file 1 [file SupplementaryFile1.docx]

Supplementary Material

# 1 Supplementary Figures and Tables

## 1.1 Supplementary Figure

**Supplementary Figure 1.** Relative mRNA expression level of inflammatory cytokine IL-6. Statistical significance among the groups was assessed by one-way analysis of variance (ANOVA) followed by Tukey’s post hoc test. Comparison with the control group, #### p < 0.0001. Comparison with the IMQ group, **** p < 0.0001.

## 1.2 Supplementary Tables

**Supplementary Table 1. PASI scoring criteria in the IMQ-induced psoriasis mouse model.**

| **Scores** | **Severity** | **Erythema** | **Thickness** | **Scale** |
| --- | --- | --- | --- | --- |
| 0 | None | None | None | None |
| 1 | Slight | Dander with a light red | Slight skinfold at the edges | Part of the skin was covered by scales |
| 2 | Moderate | The edge of the plaque was red in color | Moderate thickening | Majority of the skin was covered by sheeted scales |
| 3 | Severe | The plaque was deep red | Obvious thickening | Almost all skin was covered by thick and layered scales |
| 4 | Extremely severe | Extremely red | Extremely thickening | The whole skin was covered by especially thick and layered scales |

**Supplementary Table 2. Primers used in experiments.**

| **Gene** | **Primer (F; R)** |
| --- | --- |
| *β-actin* | GCTGTGCTATGTTGCCCTAGACTTC; GGAACCGCTCATTGCCGATAGTG |
| *TNF-α* | CCCAAAGGGATGAGAAGTTCC; GCTACAGGCTTGTCACTCGAA |
| *IL-1β* | TGCCACCTTTTGACAGTGATG; ATGTGCTGCTGCGAGATTTG |
| *IL-17A* | GCTGACCCCTAAGAAACCCC; GAAGCAGTTTGGGACCCCTT |
| *IL-6* | GACTGGGGATGTCTGTAGCTC; CAACTGGATGGAAGTCTCTTGC |

**Supplementary Table 3. Daily total PASI scores (Mean ± SD) .**

| **Time (days)** | **Control group** | **IMQ group** | **DEX group** | **RPE-L gruop** | **RPE-H group** | **PPH-L gruop** | **PPH-H group** |
| --- | --- | --- | --- | --- | --- | --- | --- |
| 1 | 0.00±0.00 | 0.00±0.00 | 0.00±0.00 | 0.00±0.00 | 0.00±0.00 | 0.00±0.00 | 0.00±0.00 |
| 2 | 0.88±0.35 | 1.00±0.93 | 1.88±0.99 | 0.88±0.35 | 1.13±0.35 | 0.75±0.46 | 1.25±1.04 |
| 3 | 0.88±0.35 | 4.88±0.64 #### | 4.00±0.76 | 4.13±0.83 | 4.25±0.89 | 4.00±0.53 | 4.25±1.04 |
| 4 | 1.00±0.00 | 8.63±0.74 #### | 7.75±0.46 | 8.13±0.83 | 8.38±0.74 | 7.50±1.20 | 8.00±1.41 |
| 5 | 1.00±0.00 | 9.63±0.52 #### | 9.13±0.83 | 9.75±0.46 | 9.13±1.36 | 8.63±0.74 | 9.38±0.74 |
| 6 | 1.00±0.00 | 10.38±0.52 #### | 10.13±0.64 | 10.00±0.53 | 10.25±0.46 | 10.63±0.74 | 10.38±0.52 |
| 7 | 1.00±0.00 | 9.38±1.19 #### | 7.50±1.20 | 7.38±1.60 | 9.25±1.04 | 9.63±0.92 | 8.38±1.06 |
| 8 | 1.00±0.00 | 9.13±1.13 #### | 8.00±0.00 | 6.75±1.04 **** | 7.00±0.93 **** | 7.75±0.89 * | 7.63±1.06 * |
| 9 | 1.38±0.52 | 7.25±0.89 #### | 6.88±0.83 | 6.38±0.92 | 6.25±0.89 * | 6.50±1.41 | 6.00±0.53 * |
| 10 | 1.38±0.52 | 6.88±0.64 #### | 6.63±1.06 | 6.38±1.60 | 5.25±0.89 * | 5.88±0.83 | 6.50±0.93 |
| 11 | 1.38±0.52 | 7.75±0.71 #### | 6.88±0.83 | 5.63±1.06 ** | 5.50±0.93 *** | 5.50±1.41 *** | 4.38±1.06 **** |
| 12 | 1.38±0.52 | 7.63±1.06 #### | 5.63±0.52 *** | 4.88±0.83 **** | 4.25±1.28 **** | 4.75±0.71 **** | 4.88±1.13 **** |
|  | Comparision with the Control group, ####p<0.0001. | | Comparision with the IMQ group, * p < 0.05, ** p < 0.01, *** p < 0.001, **** p < 0.0001. | | | | |

Data are presented as mean ± SD (n=8). For each day, statistical significance among the groups was assessed by one-way analysis of variance (ANOVA) followed by Tukey’s post hoc test. Comparison with the Control group on the same day: #### p < 0.0001. Comparison with the IMQ group on the same day: * p < 0.05, ** p < 0.01, *** p < 0.001, **** p < 0.0001.

**Supplementary Table 4. Daily Erythema scores (Mean ± SD) .**

| **Time (days)** | **Control group** | **IMQ group** | **DEX group** | **RPE-L gruop** | **RPE-H group** | **PPH-L gruop** | **PPH-H group** |
| --- | --- | --- | --- | --- | --- | --- | --- |
| 1 | 0.00±0.00 | 0.00±0.00 | 0.00±0.00 | 0.00±0.00 | 0.00±0.00 | 0.00±0.00 | 0.00±0.00 |
| 2 | 0.00±0.00 | 0.13±0.35 | 0.50±0.53 | 0.13±0.35 | 0.13±0.35 | 0.25±0.46 | 0.38±0.52 |
| 3 | 0.00±0.00 | 1.38±0.52 #### | 1.38±0.52 | 1.38±0.52 | 1.38±0.52 | 1.38±0.52 | 1.13±0.35 |
| 4 | 0.00±0.00 | 2.88±0.64 #### | 2.63±0.52 | 2.88±0.35 | 2.75±0.46 | 2.50±0.53 | 2.75±0.46 |
| 5 | 0.00±0.00 | 2.88±0.35 #### | 2.88±0.35 | 3.13±0.35 | 2.88±0.35 | 2.88±0.64 | 2.88±0.35 |
| 6 | 0.00±0.00 | 3.00±0.00 #### | 3.13±0.64 | 3.00±0.00 | 3.13±0.35 | 3.25±0.46 | 3.00±0.00 |
| 7 | 0.00±0.00 | 3.25±0.71 #### | 3.13±0.35 | 2.75±0.46 | 2.88±0.35 | 2.88±0.35 | 2.50±0.53 * |
| 8 | 0.00±0.00 | 3.75±0.46 #### | 3.50±0.53 | 2.50± 0.53 **** | 2.50±0.53 **** | 3.00±0.00 ** | 2.50±0.53 **** |
| 9 | 0.13±0.35 | 3.38±0.52 #### | 3.38±0.52 | 3.13±0.64 | 2.75±0.46 | 3.25±0.46 | 2.25±0.46 *** |
| 10 | 0.00±0.00 | 3.88±0.35 #### | 3.50±0.53 | 3.25± 0.89 | 2.50±0.76 ** | 3.13±0.83 | 3.25±0.71 |
| 11 | 0.00±0.00 | 3.88±0.35 #### | 4.00±0.00 | 3.38±0.52 | 2.75±0.46 *** | 3.38±0.74 | 2.38±0.52 **** |
| 12 | 0.00±0.00 | 3.75±0.46 #### | 4.00±0.00 | 2.63±0.92 ** | 2.00±0.93 **** | 2.50±0.53 **** | 2.50±0.53 **** |
|  | Comparisons with the control group. | | Comparisons with the IMQ group. | | | | |

Data are presented as mean ± SD (n=8). For each day, statistical significance among the groups was assessed by one-way analysis of variance (ANOVA) followed by Tukey’s post hoc test. Comparison with the Control group on the same day: #### p < 0.0001. Comparison with the IMQ group on the same day: * p < 0.05, ** p < 0.01, *** p < 0.001, **** p < 0.0001.

**Supplementary Table 5. Daily Thickness scores (Mean ± SD) .**

| **Time (days)** | **Control group** | **IMQ group** | **DEX group** | **RPE-L gruop** | **RPE-H group** | **PPH-L gruop** | **PPH-H group** |
| --- | --- | --- | --- | --- | --- | --- | --- |
| 1 | 0.00±0.00 | 0.00±0.00 | 0.00±0.00 | 0.00±0.00 | 0.00±0.00 | 0.00±0.00 | 0.00±0.00 |
| 2 | 0.88±0.35 | 0.63±0.52 | 1.13±0.35 | 0.75±0.46 | 1.00±0.00 | 0.50±0.53 | 1.00±0.53 |
| 3 | 0.88±0.35 | 2.13±0.64 # | 1.88±0.64 | 2.00±0.93 | 1.63±0.74 | 1.75±0.71 | 2.25±0.89 |
| 4 | 1.00±0.00 | 3.13±0.35 #### | 3.13±0.35 | 2.88±0.35 | 3.00±0.00 | 2.88±0.35 | 2.75±0.46 |
| 5 | 1.00±0.00 | 3.00±0.00 #### | 3.00±0.00 | 3.00±0.00 | 3.00±0.00 | 3.00±0.00 | 2.88±0.35 |
| 6 | 1.00±0.00 | 3.13±0.35 #### | 3.00±0.00 | 3.00±0.00 | 3.00±0.00 | 3.13±0.35 | 3.00±0.00 |
| 7 | 1.00±0.00 | 2.88±0.35 #### | 2.25±0.71 | 2.25±0.46 | 3.25±0.71 | 3.13± 0.64 | 2.50±0.53 |
| 8 | 1.00±0.00 | 3.38±0.74 #### | 3.38±0.52 | 2.75±0.46 | 3.25±0.46 | 3.13± 0.35 | 3.25±0.71 |
| 9 | 1.38±0.52 | 3.00±0.53 #### | 3.00± 0.00 | 2.50±0.53 | 3.00±0.53 | 2.63±0.52 | 2.75±0.46 |
| 10 | 1.38±0.52 | 2.75±0.46 #### | 2.63±0.52 | 2.25±0.71 | 2.75±0.46 | 2.75±0.71 | 3.00±0.53 |
| 11 | 1.38±0.52 | 3.25±0.71 #### | 2.38±0.52 | 2.13±0.64 ** | 2.88±0.64 | 2.13±0.83 * | 1.88±0.83 ** |
| 12 | 1.38±0.52 | 3.50±0.53 #### | 1.63±0.52 **** | 2.25±0.71 *** | 2.25±0.46 *** | 2.50±0.76 ** | 2.25±0.46 *** |
|  | Comparisons with the control group. | | Comparisons with the IMQ group. | | | | |

Data are presented as mean ± SD (n=8). For each day, statistical significance among the groups was assessed by one-way analysis of variance (ANOVA) followed by Tukey’s post hoc test. Comparison with the Control group on the same day: # p < 0.05, #### p < 0.0001. Comparison with the IMQ group on the same day: * p < 0.05, ** p < 0.01, *** p < 0.001, **** p < 0.0001.

**Supplementary Table 6. Daily Scales scores (Mean ± SD) .**

| **Time (days)** | **Control group** | **IMQ group** | **DEX group** | **RPE-L gruop** | **RPE-H group** | **PPH-L gruop** | **PPH-H group** |
| --- | --- | --- | --- | --- | --- | --- | --- |
| 1 | 0.00±0.00 | 0.00±0.00 | 0.00±0.00 | 0.00±0.00 | 0.00±0.00 | 0.00±0.00 | 0.00±0.00 |
| 2 | 0.00±0.00 | 0.13±0.35 | 0.25±0.46 | 0.00±0.00 | 0.00±0.00 | 0.00±0.00 | 0.13±0.35 |
| 3 | 0.00±0.00 | 1.38±0.52 #### | 0.75±0.46 | 1.00±0.00 | 1.25±0.46 | 0.88±0.64 | 1.00±0.53 |
| 4 | 0.00±0.00 | 2.75±0.46 #### | 2.13±0.35 | 2.38±0.52 | 2.75±0.46 | 2.25±0.71 | 2.63±0.74 |
| 5 | 0.00±0.00 | 3.75±0.46 #### | 3.38±0.74 | 3.50±0.76 | 3.38±0.92 | 2.88±0.83 | 3.25±0.89 |
| 6 | 0.00±0.00 | 4.00±0.00 #### | 4.00±0.00 | 3.88±0.35 | 3.88±0.35 | 4.00±0.00 | 4.00±0.00 |
| 7 | 0.00±0.00 | 3.25±1.04 #### | 2.13±0.64 | 2.38±1.19 | 3.13±0.99 | 3.63±0.52 | 3.38±0.74 |
| 8 | 0.00±0.00 | 2.00±0.76 #### | 1.13±0.35 | 1.50±0.53 | 1.25±0.71 | 1.63±0.74 | 1.75±0.71 |
| 9 | 0.00±0.00 | 0.88±0.64 # | 0.50±0.76 | 0.88±0.64 | 0.63±0.52 | 0.63±0.74 | 0.88±0.35 |
| 10 | 0.00±0.00 | 0.25±0.46 | 0.50±0.53 | 0.63±0.52 | 0.00±0.00 | 0.25±0.46 | 0.25±0.46 |
| 11 | 0.00±0.00 | 0.63±0.52 # | 0.50±0.53 | 0.25±0.46 | 0.00±0.00 * | 0.00±0.00 * | 0.00±0.00 * |
| 12 | 0.00±0.00 | 0.50±0.53 ## | 0.00±0.00 ** | 0.00±0.00 ** | 0.00±0.00 ** | 0.13±0.35 * | 0.00±0.00 ** |
|  | Comparisons with the control group. | | Comparisons with the IMQ group. | | | | |

Data are presented as mean ± SD (n=8). For each day, statistical significance among the groups was assessed by one-way analysis of variance (ANOVA) followed by Tukey’s post hoc test. Comparison with the Control group on the same day: # p < 0.01, ## p < 0.01, #### p < 0.0001. Comparison with the IMQ group on the same day: * p < 0.05, ** p < 0.01, *** p < 0.001, **** p < 0.0001.
